# Changes in Medicaid enrollment during the COVID-19 pandemic across 6 states

**DOI:** 10.1097/MD.0000000000032487

**Published:** 2022-12-30

**Authors:** Ran Sun, Becky Staiger, Antonia Chan, Laurence C. Baker, Tina Hernandez-Boussard

**Affiliations:** a Department of Medicine, Center for Biomedical Informatics Research, Stanford University School of Medicine, Stanford, CA; b Stanford University School of Medicine, Stanford, CA; c The Center for Health Policy and the Center for Primary Care and Outcomes Research, Stanford University School of Medicine, Stanford, CA; d Stanford Institute for Economic Policy Research, Stanford University, Stanford, CA; e Department of Biomedical Data Science, Stanford University School of Medicine, Stanford, CA; f Department of Surgery, Stanford University School of Medicine, Stanford, CA.

**Keywords:** COVID-19, health policy, health services research, healthcare enrollment, Medicaid

## Abstract

The coronavirus disease 2019 public health emergency (PHE) caused extensive job loss and loss of employer-sponsored insurance. State Medicaid programs experienced a related increase in enrollment during the PHE. However, the composition of enrollment and enrollee changes during the pandemic is unknown. This study examined changes in Medicaid enrollment and population characteristics during the PHE. A retrospective study documenting changes in Medicaid new enrollment and disenrollment, and enrollee characteristics between March and October 2020 compared to the same time in 2019 using full-state Medicaid populations from 6 states of a wide geographical region. The primary outcomes were Medicaid enrollment and disenrollment during the PHE. New enrollment included persons enrolled in Medicaid between March and October 2020 who were not enrolled in January or February, 2020. Disenrollment included persons who were enrolled in March of 2020 but not enrolled in October 2020. The study included 8.50 million Medicaid enrollees in 2020 and 8.46 million in 2019. Overall, enrollment increased by 13.0% (1.19 million) in the selected states during the PHE compared to 2019. New enrollment accounted for 24.9% of the relative increase, while the remaining 75.1% was due to disenrollment. A larger proportion of new enrollment in 2020 was among adults aged 27 to 44 (28.3% vs 23.6%), Hispanics (34.3% vs 32.5%) and in the financial needy (44.0% vs 39.0%) category compared to 2019. Disenrollment included a larger proportion of older adults (26.1% vs 8.1%) and non-Hispanics (70.3% vs 66.4%) than in 2019. Medicaid enrollment grew considerably during the PHE, and most enrollment growth was attributed to decreases in disenrollment rather than increases in new enrollment. Our results highlight the impact of coronavirus disease 2019 on state health programs and can guide federal and state budgetary planning once the PHE ends.

## 1. Introduction

The coronavirus disease 2019 (COVID-19) pandemic has generated dual public health and economic crises in the US. To minimize transmission of the coronavirus, many policies restricting business activities and social interactions were implemented, and many individuals curtailed their activities,^[1,2]^ which led to temporary or permanent closures of businesses and large-scale job losses.^[3,4]^ As of January 2021, there were >75 million initial unemployment insurance claims filed since the start of the public health emergency (PHE).^[5]^

Medicaid provides a valuable option for individuals facing economic challenges, particularly those experiencing job losses that lead to the loss of employer-sponsored health insurance (ESI).^[6]^ One recent estimate from Blue Cross Blue Shield Association finds that among people who lost ESI between March and October 2020, nearly 60% enrolled in Medicaid.^[7]^ Consistent with this, recent reports have shown substantial increases in Medicaid enrollment during the pandemic,^[8]^ including a Medicaid enrollment increase of 7.6 million nationally between February and November 2020.^[9]^

Increases in Medicaid enrollment may be the result of 2 separate phenomena. First, changing economic circumstances may have made people newly eligible for Medicaid and studies confirm that employment-sponsored coverage declined during the PHE.^[10,11]^ During the PHE, state Medicaid programs undertook various actions to increase accessibility and expand coverage for their vulnerable citizens.^[12]^ Second, as a response to the COVID-19 pandemic, the federal government enacted the Families First Coronavirus Response Act (FFCRA), increasing federal Medicaid match funding in exchange for states guaranteeing continuous coverage for enrollees during the PHE period.^[13]^ Responding to the FFCRA, states extended eligibility for individuals already enrolled in Medicaid who might otherwise have become ineligible.^[9,14]^ Accordingly, a recent study in a single state suggested that Medicaid enrollment during the PHE was largely due to the maintenance of continuous coverage rather than new enrollees due to unemployment^[15]^ and another study found a significant increase in enrollment for safety net programs during the PHE.^[16]^ However, analyses have not assessed the relative importance of increased new enrollment as compared with reduced disenrollment as drivers of increased total enrollment. A better understanding of this would be valuable for demonstrating the extent of the shift of insurance coverage to Medicaid and the impact of FFCRA on encouraging continuous coverage access during the pandemic. Relatedly, it could shed light on patterns of “churning,” in which people may cycle on and off Medicaid in ways that indicate unstable coverage during the pandemic.

Understanding the enrollment patterns would contribute to a better understanding of overall health insurance coverage trends during the pandemic, as recently suggested by Glied and Swartz.^[17]^ In this study, we examined enrollment trends and populations during the PHE compared to trends in 2019. A better understanding of who enrolls in Medicaid during the pandemic and through which coverage pathway provides insight on the shift of insurance coverage during the PHE, further healthcare utilization and Medicaid expenditure, thus assisting in federal and state budgetary planning and policy development in the future.

## 2. Methods

### 2.1. Study design

This retrospective descriptive study used Medicaid enrollment data from 6 states spanning a broad geographical region. Changes were examined regarding new enrollment and disenrollment, and enrollee characteristics between March and October 2020 compared to the same period in 2019, based on similar literature.^[18]^ To our knowledge, none of our sample states had any policy changes regarding enrollment in 2019 that might bias their relevance as a baseline measure of non-pandemic enrollment. This study received the approval from the institute’s Institutional Review Board.

### 2.2. Data source

De-identified Medicaid enrollment data between January 2015 and October 2020 for 6 states were used. These 6 states provided their data for research purposes. The data include all Medicaid enrollment in the states, capturing both fee-for-service and Medicaid managed care enrollment. Children’s Health Insurance Program enrollees were not included. The data are collected by Health Management Systems and managed by the Digital Health Cooperative Research Centre. Due to contractual agreements, the included states cannot be named. The included states represent diverse geographical regions, including 3 southwestern states, 1 midwestern state, and 2 southeastern states. Of the 6 states in our sample, 5 expanded Medicaid coverage under the Affordable Care Act (ACA). This study followed the Strengthening the Reporting of Observational Studies in Epidemiology reporting guidelines.

### 2.3. Population

Using the coverage start and end dates recorded for each Medicaid enrollee, 3 mutually exclusive cohorts were constructed in 2019 and 2020: individuals who were observed on both March 1 and October 31 (“continuous enrollees”); individuals who were not observed on March 1 but were observed on October 31 (“new entrants”); individuals observed on March 1 but not on October 31 (“disenrollees”). We also constructed a fourth cohort, which may help us shed light on short-term enrollment and disenrollment patterns. Specifically, we identified individuals who were enrolled at some point between March and October but who were not observed on either March 1 or October 31 (“short-term enrollees”).

### 2.4. Primary outcomes

Our primary outcome of interest was the change in Medicaid enrollment during the PHE. Enrollment was measured on a per month basis. Individuals enrolled at any time during a given month were counted in the total monthly enrollment.

### 2.5. Variables

For members of our new entrant cohorts, we identified the enrollees who did not have a previous Medicaid enrollment record in the same state the prior 4 years, “first-time” new entrants. Our lookback period for identifying this group in 2019 started in 2015, and our lookback period for the 2020 cohort started in 2016. The 5-year lookback period included all the available data. We also identified enrollee death, which was available in the enrollment files as death date.

We compiled information about enrollee age and sex for all our cohorts. We assigned each enrollee to one of the following age groups: ≤18, 19 to 26, 27 to 44, 45 to 64, and ≥65. We had complete information on age for each person, and sex was missing for <0.02% of the study cohort. Enrollee race and ethnicity were not consistently recorded across states. In the 3 southwest states, ethnicity appeared to be consistently reported as a binary variable (Hispanic or non-Hispanic of any race). In the other states, race and ethnicity were inconsistently, and sometimes incompletely, reported. For members of our cohorts from the 3 southwestern states, we constructed an indicator of Hispanic ethnicity. We benchmarked the accuracy of our calculated composition of Hispanic and non-Hispanic enrollees with publicly available reports.^[19,20]^ About 0.3% of the persons observed between March and October in 2019 and 2020 had missing values on ethnicity.

Finally, we compiled information on Medicaid eligibility. The enrollment data includes eligibility information, which differed across states. We identified 2 states that included consistent categories. Using this complete eligibility information from 2 states, we constructed a binary indicator that identified individuals eligible as “low-income adults.” This category includes adults 16 to 64 years of age with income up to 138% of the federal poverty level who gained access to Medicaid because of the ACA. This category did not include enrollees who were Medicare eligible, pregnant, and parents or caretakers of a child.

### 2.6. Statistical analysis

Using enrollees’ coverage start and end dates, we calculated total Medicaid enrollment for each month in 2019 and 2020. In monthly enrollment comparisons between 2019 and 2020, we determined the percent change in Medicaid enrollment relative to enrollment in January each year. We then compared changes in enrollment between March and October of 2019 and 2020, and attributed changes to the new entrant and disenrollee categories, a methodology that has been used in prior studies examining changes in healthcare trends during the pandemic.^[21]^ We further compared differences in the number of short-term enrollees, first-time new entrants, and disenrollees associated with death in the 2 years.

We examined changes in the distribution of individuals by age, sex, ethnicity, and “low-income adult” eligibility category using Pearson chi-square tests. We set the significance level at 0.05. All analyses were performed using R statistical software (R version 3.5.1 and R Studio 1.1.456).

## 3. Results

**Figure 1.**
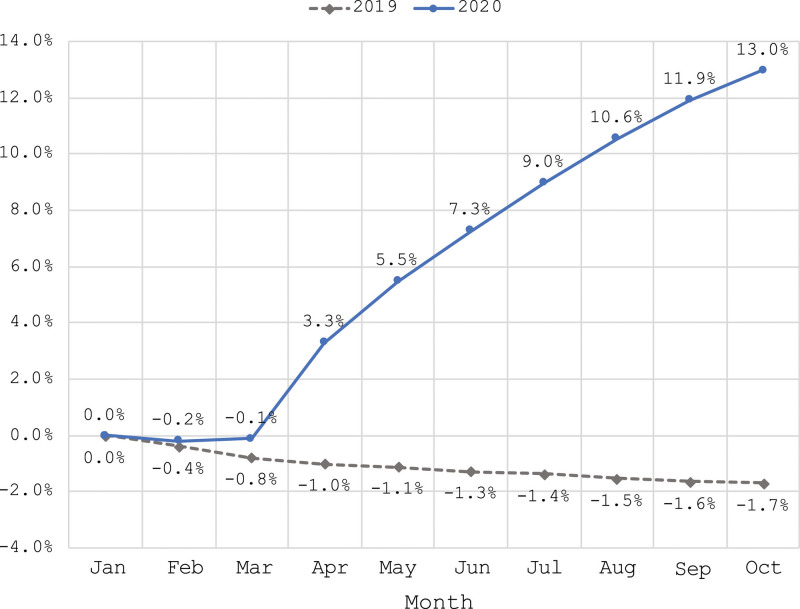
Percent change in monthly measured Medicaid enrollment relative to January in 2019 versus 2020.

This study included approximately 8.5 million unique Medicaid members in total each year, accounting for nearly 11% of the total US Medicaid population (eTab S4, Supplemental Digital Content, http://links.lww.com/MD/I243)). The percent change in monthly Medicaid enrollment in 2019 and 2020 benchmarked to each state’s enrollment in January of that year is presented in Figure 1. During the PHE, Medicaid enrollment increased substantially, with a notable increasing trend starting in April 2020 and continuing through October. Across all the states, enrollment was 13.0% higher in October 2020 than in January 2020. In 2019, there was a slight declining trend in enrollment. Monthly Medicaid enrollment by state is presented in the supplement (eTab S1, Supplemental Digital Content, http://links.lww.com/MD/I240). Based on the pattern observed in Figure 1, it appears that March 2020 enrollment will provide a suitable baseline for tracking changes during the pandemic period. We thus focus further analysis on changes between March and October of 2019 and 2020.

**Figure 2.**
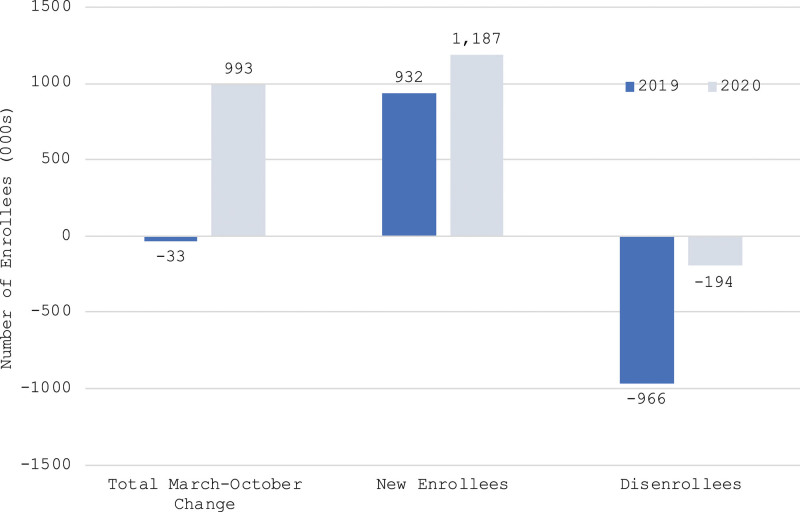
Comparison of number of new entrants and disenrollees between 2019 and 2020 (figures in thousands of enrollees).

Figure 2 shows that enrollment grew substantially more between March and October of 2020 than over the same period in 2019. Whereas enrollment in the 6 study states declined by about 33,000 between March and October 2019, it grew by 993,000 (13.6%) in 2020. “Relative enrollment growth” in 2020 compared to 2019 was thus about 1026,000, calculated as the change in 2020 relative to the change over the same time in 2019 (993,000 + 33,000).

The March to October change in enrollment was equal to the number of new entrants minus the number of disenrollees. Figure 2 shows that the larger March to October growth in 2020 was partly because there were more new Medicaid entrants in 2020. Between March and October 2020, our study states saw about 1187,000 new entrants, about 254,000 (27%) more than in 2019. The overall March to October enrollment growth was also due to a dramatic decline in the number of disenrollees. Whereas about 772,000 people in our study states disenrolled between March and October 2019, only about 194,000 people did so in 2020, a decline of about 80%. These results show that lower rates of disenrollment were much more important than higher rates of new enrollment in explaining 2020’s higher relative enrollment growth. The new enrollment difference only accounts for about 25% of the relative enrollment increase (1187,000 − 932,000 = 255,000, divided by relative enrollment increase of 1026,000). The remaining 75% of the net enrollment growth in 2020 was due to lower disenrollment in 2020.

Table 1 provides additional information about enrollment changes in our cohorts between the 2 years. Results in Table 1 show that new Medicaid enrollees in 2020 were somewhat more likely to have had Medicaid previously. Whereas in 2019, 46% of new entrants to Medicaid had been enrolled in the prior 4 years (429,000 of 932,000 new entrants), in 2020, this share rose to 52% (607,000 of 1187,000).

**Table 1:** March to October changes in enrollment, overall and by category, 2019 and 2020.

	2019 enrollees (000s)	2020 enrollees (000s)	Difference between 2020 and 2019
Enrollees, March 1	7351	7285	−66
Enrollees, October 31	7317	8279	961
Change from March to October	−33	993	1027
Percent change	−0.5%	13.6%	14.1%
By category			
New entrants (March–October)	932	1187	255
Medicaid prior 4 yr	503	580	77
Had Medicaid prior 4 yr	429	607	178
Disenrollees (March–October)	966	194	−772
With March to October death date	48	59	11
Without March to October death date	918	134	−783
Continuous enrollees	6385	7092	707
Short-term enrollees	178	28	−150

New entrants are individuals not enrolled on March 1 but enrolled on October 31. Disenrollees are individuals enrolled on March 1 but not on October 31. Continuous enrollees are individuals enrolled on both March 1 and October 31. Short-term enrollees are individuals not enrolled either on March 1 or October 31 but enrolled at any point in between.

Further detail on disenrollees in Table 1 shows that there was somewhat more disenrollment due to death in 2020 than in 2019. The year 2020 saw about 11,000 additional disenrollments with a death date between March and October than 2019. About 9000 of the incremental disenrollments associated with death were in the ≥65 age group, and about 2000 were among adults aged 19 to 64 (eTab S2, Supplemental Digital Content, http://links.lww.com/MD/I241).

Table 1 also shows that there were substantially fewer “short-term” enrollees who first enrolled after March but disenrolled before October in 2020 than in 2019. Whereas there were about 178,000 such enrollees in 2019, there were only 28,000 in 2020, a decline of about 84%. This is consistent with less disenrollment in general in 2020. There were also more continuous enrollees in 2020 than in 2019, consistent with the lower disenrollment rates.

The top panel of Table 2 shows how the numbers of new entrants and disenrollees were distributed by age and sex categories. All differences between 2019 and 2020 distributions were statistically significant given the large sample size (*P* < .001). Adults aged 19 to 64 account for the largest shares of the 2019 to 2020 swings in new enrollment and disenrollment. New enrollment by adults aged 19 to 64 made up about 55% of all new enrollment in 2020, compared with 48% in 2019. Looking at it another way, total new enrollment in 2020 was about 255,000 higher than in 2019, 77% of which is accounted for by adults aged 19 to 64. Within this larger age group, adults aged 27 to 44 were the largest subgroup and made up more than half of the new enrollment. New enrollment among people <18 years was only modestly higher in 2020 compared to 2019, and new enrollment by people aged ≥65 was nearly the same. Disenrollment in 2020 was much lower in both the age 19 to 64 and the age ≤18 groups. Disenrollment also declined among those aged ≥65, but much less precipitously. New enrollment and disenrollment patterns were very similar for men and women. The comparison of demographic and the eligibility category for continuous and short-term enrollees is presented in the supplement (eTab S3, Supplemental Digital Content, http://links.lww.com/MD/I242).

**Table 2:** Characteristics of new entrants and disenrollees from March 1 to October 31 in 2019 versus 2020.

	New entrants (000s)				Disenrollees (000s)			
	2019		2020		2019		2020	
	N	(%)	N	(%)	N	(%)	N	(%)
Using 6-state sample								
N	932		1187		966		194	
Age								
0–18	417	(45%)	476	(40%)	381	(39%)	63	(33%)
19–64	449	(48%)	647	(55%)	507	(52%)	80	(41%)
19–26	115	(12%)	151	(13%)	155	(16%)	19	(10%)
27–44	220	(24%)	336	(28%)	231	(24%)	32	(17%)
45–64	114	(12%)	160	(13%)	121	(13%)	29	(15%)
≥65	66	(7%)	64	(5%)	78	(8%)	51	(26%)
Sex								
Female	519	(56%)	667	(56%)	535	(55%)	104	(54%)
Male	413	(44%)	519	(44%)	431	(45%)	89	(46%)
Using 3-state sample								
N	674		888		666		120	
Hispanic ethnicity								
Hispanic	218	(32%)	305	(34%)	224	(34%)	36	(30%)
Non-Hispanic	455	(68%)	581	(65%)	440	(66%)	84	(70%)
Using 2-state sample								
N	232		256		265		56	
Eligibility category								
Low-income adult	90	(39%)	111	(44%)	103	(39%)	16	(29%)
Other	142	(61%)	145	(56%)	161	(61%)	40	(71%)

Data for sex distribution excludes <0.1% cases reporting unknown sex. Data for Hispanic ethnicity distribution excludes <0.6% of cases reporting unknown ethnicity. New entrants are members not observed on March 1 but on October 31. Disenrollees are members observed on March 1 but not on October 31. All differences between 2019 and 2020 were significant (*P* < .001).

Table 2 also shows that in the 3 states where we could observe Hispanic ethnicity, people of Hispanic ethnicity made up a larger share of the new entrants in 2020 and were also comparatively less likely to disenroll in 2020 than in 2019. In 2 states where we could observe the aid category, individuals enrolled through the “low-income adults” aid category in 2020 accounted for 44% of all new enrollment, compared with 39% in 2019. Also, disenrollees in 2020 were less likely from this eligibility group; 39% of disenrollees belonged to this group in 2019, and this share decreased to 29% in 2020.

## 4. Discussion

From 2017 to 2019, national Medicaid enrollment declined by 2.6%. The COVID-19 pandemic reversed this course abruptly, with Medicaid enrollment increasing by 15.5% from February 2020 relative to April 2021. While existing research points to massive job loss as one primary driver of this increased enrollment, this study found that approximately three-fourths of Medicaid enrollment growth could be traced to declines in the rate of disenrollment relative to 2019, with only one quarter explained by higher rates of new enrollment during the March to October study period. Reduced disenrollment rates may be attributable to beneficiaries’ sustained Medicaid eligibility due to job loss or other economic circumstances, or to the enrollment protections offered by the FFCRA.

The fact that new enrollment played a smaller role than disenrollment may be surprising, given expectations that economic hardships caused by the pandemic would lead to widespread new enrollment in Medicaid.^[6,22]^ However, more recent reports suggest that loss of ESI was less widespread than originally expected^[7]^; and only one-third of people who lost ESI would obtain coverage from Medicaid.^[23]^ Additionally, more stringent Medicaid eligibility requirements, particularly in states that had not expanded Medicaid, likely kept enrollment lower than would have been expected based on need.^[21]^ It is also important to note that, while this analysis emphasizes the major role of the decreased number of disenrollees on the enrollment growth, we still observe a 27% increase in new enrollment in 2020 compared to 2019, which is non-negligible and possibly reflects changes in the economy.

Lower rates of disenrollment could reflect a decline in opportunities for Medicaid enrollees to obtain income increases or insurance from other sources during the pandemic. They also seem likely to reflect the effects of the FFCRA, which aimed to secure continuous coverage for Medicaid recipients. Specifically, the FFCRA prevents state Medicaid agencies from terminating coverage for enrollees during the PHE in exchange for increased federal cost-sharing.^[13]^ Though we are not able to fully separate effects of the FFCRA from effects of the economic downturn that may have independently led to declines in disenrollment, it is plausible that the economic downturn itself may have had effects of similar magnitude on new enrollment and disenrollment. Thus, the larger overall decline in disenrollment may suggest an impact of the FFCRA over and above that of just the economic downturn. We also found many fewer short-term enrollees in 2020 compared to 2019, further consistent with observed declines in disenrollment.

In this study, there were higher rates of new enrollment among people aged 19 to 64 than among older and younger groups. Within that group, those aged 27 to 44 were the most different between 2019 and 2020, similar to findings from a Census Bureau’s Experimental Household Pulse Survey.^[24]^ Another study found that new Medicaid enrollees during the PHE had lower rates of health care utilization compared to pre-COVID enrollees.^[25]^ This may reflect that people in this age group were particularly impacted financially by the pandemic but did not have a pent-up demand for healthcare services, as was noted by Taubman et al^[26]^ Our finding coincides with reports of unemployment rates peaking during the pandemic among young adults, with unemployment rates ranging from 29% in the youngest age category to 12% in older adults.^[27,28]^ As this study uses data from full state Medicaid programs, the results build on and substantiate existing literature regarding the effect of federal programs targeting Medicaid disenrollment during the PHE.

We found comparatively higher rates of new enrollment among people of Hispanic ethnicity, also consistent with reports that the pandemic may have had disproportionate effects on this population.^[29]^ The study showed a higher share of new entrants enrolled via the ACA-expanded “low-income adults” category, highlighting the importance of the ACA expansion on gaining coverage and accessing healthcare services during the economic downturn, particularly among populations at higher risks of COVID-19.^[30]^ Access to Medicaid during the pandemic allow vulnerable groups to access care during their financial hardship.

The decline in disenrollment we observe suggests the value of efforts to preserve eligibility during the pandemic. This finding builds on prior work that Wisconsin Medicaid enrollment during the PHE was predominantly due to the continued coverage rather than new enrollees.^[15]^ Our work shows similar results across multiple states. To the extent to which the reduction of enrollment is due to policy changes like FFCRA, policies appear to have reduced instability in Medicaid access and promote continuity of coverage during this time. This appears to have disproportionately benefitted young working-age adults, people of Hispanic ethnicity, and the ACA-expanded adults whose income is below the 138% federal poverty level.

The increasing number of Medicaid enrollees in 6 states studied highlights the importance of the program, especially during the PHE. Understanding the composition of the persons enrolled in Medicaid during the pandemic may have implications for the mix of services paid by Medicaid, as well as the success of outreach efforts. Anticipating who will be enrolling into Medicaid because of the PHE and understanding their healthcare utilization may be valuable for state Medicaid agencies and budget officials as they plan for an unforeseeable future.

## 5. Limitations

There are several limitations to this study. First, our data is limited to 6 states which might not reflect nationwide trends in Medicaid enrollment. However, the states are diverse and from different geographical areas (as we show in eTab S4, Supplemental Digital Content, http://links.lww.com/MD/I243). Second, the eligibility category and ethnicity information are available from only a few states in our sample, which may limit the generalizability of our findings for these measures. Third, our ability to identify first-time new entrants is limited to them enrolling for the first time within the same state. Enrollees are assigned unique state-level identifiers, but these are not valid across states. Therefore, we may incorrectly characterize enrollees as “first-time” when they were in fact previously enrolled in another state’s Medicaid program. Fourth, we did not track individual patients from 2019 to 2020, therefore we do not know what percent of the 2019 cohort is captured in the 2020 cohort. However, this does not affect the overall interpretation of the findings. Finally, there could be differences in Medicaid enrollment patterns even in the absence of COVID that could bias our results.

## 6. Conclusions

The Medicaid population grew considerably during the PHE in the 6 states examined. This was mainly due to the dramatic decrease in the number of disenrollees during the PHE. Medicaid enrollment growth significantly increased among the working-aged, financially needy population, and Medicaid churning decreased. These results provide important insights related to the impact of the PHE on the Medicaid program and highlight the need for policies targeting continuous enrollment that extend beyond the PHE. Policy-makers and key stakeholders can leverage this evidence to guide program planning when the PHE ends and to ensure coverage for affected populations in the future.

## Author contributions

Dr Tina Hernandez-Boussard had full access to all the data in the study and takes responsibility for the integrity of the data and the accuracy of the data analysis.

**Administrative, technical, or material support:** Tina Hernandez-Boussard.

**Conceptualization:** Laurence C. Baker, Tina Hernandez-Boussard.

**Data curation:** Ran Sun, Antonia Chan.

**Data interpretation:** All authors.

**Formal analysis:** Ran Sun, Antonia Chan.

**Funding acquisition:** Tina Hernandez-Boussard.

**Methodology:** Becky Staiger, Antonia Chan, Laurence C. Baker.

**Project administration:** Tina Hernandez-Boussard.

**Resources:** Tina Hernandez-Boussard.

**Statistical analysis:** Ran Sun.

**Supervision:** Laurence C. Baker, Tina Hernandez-Boussard.

**Validation:** Tina Hernandez-Boussard.

**Writing – original draft:** Ran Sun, Becky Staiger, Antonia Chan.

**Writing – review & editing:** Becky Staiger, Laurence C. Baker, Tina Hernandez-Boussard.

## Supplementary Material
