# Supplementary material for: Changes in Medicaid enrollment during the COVID-19 pandemic across 6 states
Source: Medicine (Baltimore). 2022 Dec 30;101(52):e32487. doi: 10.1097/MD.0000000000032487 (PMC9803338; doi:10.1097/MD.0000000000032487)
Supplement: Supplementary file 1 [file medi-101-e32487-s001.pdf]

eTable 1. Total Monthly Medicaid Enrollees Between January and October in 2019 and 2020 by State

Yr-Mon	2020-01	2020-02	2020-03	2020-04	2020-05	2020-06	2020-07	2020-08	2020-09	2020-10
**State 1**	655679	653329	656759	681519	698489	711095	724215	737940	749950	759741
**State 2**	838901	838650	840649	851171	861105	868348	876353	883815	888423	887626
**State 3**	1185449	1178939	1171636	1207437	1226877	1248400	1268662	1289164	1307205	1322313
**State 4**	317588	325084	331474	338428	343629	348279	353491	358997	363368	365770
**State 5**	497107	496530	492868	499081	505262	511227	516728	522078	526604	529858
**State 6**	3835079	3821807	3827213	3992690	4096166	4176285	4248374	4311757	4367465	4415646
**2019**	**2019-01**	**2019-02**	**2019-03**	**2019-04**	**2019-05**	**2019-06**	**2019-07**	**2019-08**	**2019-09**	**2019-10**
**State 1**	671671	667993	665733	665706	664069	663035	664476	663360	662400	660944
**State 2**	841808	840527	839512	838478	836091	833563	833460	833687	835795	835864
**State 3**	1236174	1231224	1225191	1211538	1210046	1207216	1199664	1199500	1200561	1196674
**State 4**	274107	275628	276814	278086	279321	280248	271024	265782	268428	270683
**State 5**	513275	513147	509277	507947	506490	504579	505174	504380	502074	501230
**State 6**	3913399	3892797	3873381	3873814	3870373	3866532	3874006	3869280	3859440	3858439

NOTE Data includes individuals enrolled at any time in the month.
