# Supplementary material for: Changes in Medicaid enrollment during the COVID-19 pandemic across 6 states
Source: Medicine (Baltimore). 2022 Dec 30;101(52):e32487. doi: 10.1097/MD.0000000000032487 (PMC9803338; doi:10.1097/MD.0000000000032487)
Supplement: Supplementary file 2 [file medi-101-e32487-s002.pdf]

eTable 2. Medicaid Enrollee Counts by Continuity Cohort and by Age Group in 2019 and 2020

	Age 0-18		Age 19-64		Age 65 and over	
	2019	2020	2019	2020	2019	2020
**March Enrollment**						
Enrollees, March 1	3,549,131	3,466,059	2,937,463	2,935,746	864,245	883,464
Continuous enrollees	3,168,389 (89.3)	3,402,788 (98.2)	2,430,776 (82.8)	2,855,986 (97.3)	785,989 (91.0)	832,911 (94.3)
Disenrollees	380,742 (10.7)	63,271 (1.8)	506,687 (17.2)	79,760 (2.7)	78,256 (9.1)	50,553 (5.7)
Disenroll due to death	764 (<0.1)	760 (<0.1)	14,771 (0.5)	16,940 (0.6)	32,426 (3.8)	41,398 (4.7)
**October Enrollment**						
Enrollees, October 31	3,585,576	3,878,908	2,879,578	3,502,853	852,289	896,825
Continuous enrollees	3,168,389 (88.4)	3,402,788 (87.7)	2,430,776 (84.4)	2,855,986 (81.5)	785,989 (92.2)	832,911 (92.9)
New Entrants	417,187 (11.6)	476,120 (12.3)	448,802 (15.6)	646,867 (18.5)	66,300 (7.8)	63,914 (7.1)
First-time enrollees	250,504 (7.0)	253,553 (6.5)	206,638 (7.2)	280,539 (8.0)	45,805 (5.4)	45,567 (5.1)
**Change March 1 to October 31**	36,445	412,849	-57,885	567,107	-11,956	13,361
**Percent change**	1.0%	12.1%	-2.0%	19.8%	-1.4%	1.8%

NOTE Continuous enrollees are members observed on both March 1 and October 31. Disenrollees are members observed on March 1 but not on October 31. New entrants are members observed on March 1 but on October 31. First-time enrollees are entrants who enrolled in Medicaid with no enrollment record in the past 48 months.
