# Supplementary material for: Changes in Medicaid enrollment during the COVID-19 pandemic across 6 states
Source: Medicine (Baltimore). 2022 Dec 30;101(52):e32487. doi: 10.1097/MD.0000000000032487 (PMC9803338; doi:10.1097/MD.0000000000032487)
Supplement: Supplementary file 3 [file medi-101-e32487-s003.pdf]

eTable 3. Characteristics of Continuous and Short-term Enrollees From March 1 to October 31 in 2019 vs. 2020

	Continuous Enrollees (000s)				Short-term Enrollees (000s)			
	2019		2020		2019		2020	
	N	(%)	N	(%)	N	(%)	N	(%)
Using 6 State Sample								
N	6385		7092		178		28	
Age								
0-18	3168	(50%)	3403	(48%)	30	(17%)	8	(27%)
19-64	2431	(39%)	2856	(41%)	138	(77%)	17	(62%)
19-26	492	(8%)	619	(9%)	293	(16%)	4	(13%)
27-44	1059	(17%)	1267	(18%)	767	(43%)	8	(29%)
45-64	881	(14%)	970	(14%)	319	(18%)	6	(20%)
>=65	786	(12%)	833	(12%)	10	(5%)	3	(12%)
Sex								
Female	3519	(55%)	3908	(55%)	108	(61%)	15	(54%)
Male	2866	(45%)	3183	(45%)	697	(39%)	13	(45%)
Using 3 State Sample								
N	4679		517		148		16	
Hispanic Ethnicity								
Hispanic	1615	(35%)	177	(34%)	53	(36%)	6	(38%)
Non-Hispanic	3051	(65%)	339	(66%)	95	(64%)	10	(62%)
Using 2 State Sample								
N	1797		1953		23		7	
Eligibility Category								
Low-income adult	553	(31%)	619	(32%)	12	(50%)	3	(44%)
Other	1244	(%)	1334	(68%)	12	(50%)	4	(56%)

NOTE Data for sex distribution excludes < 0.1% cases reporting unknown sex. Data for Hispanic ethnicity distribution excludes < 0.3% of cases reporting unknown ethnicity. Continuous enrollees are members observed on both March 1 and October 31. Short-term enrollees are members not observed either on March 1 or October 31. All differences between 2019 and 2020 were significant (p < 0.001).
