# Supplementary material for: Changes in Medicaid enrollment during the COVID-19 pandemic across 6 states
Source: Medicine (Baltimore). 2022 Dec 30;101(52):e32487. doi: 10.1097/MD.0000000000032487 (PMC9803338; doi:10.1097/MD.0000000000032487)
Supplement: Supplementary file 4 [file medi-101-e32487-s004.pdf]

eTable 4. State Demographics

STATE	1	2	3	4	5	6
Mean Age	44	38.5	39.1	35.7	39.9	37
Metropolitan Area (%)	72.2	82.1	76.5	87.4	75.3	81.7
Bachelors or higher (%)	25.8	29.5	25.7	28	25.8	27.5
County Median Household Income ($)	56,668	59,273	54,400	55,504	54,321	56,479
Unemployment rate(%)	4.32	4.02	4.49	3.85	4.42	4.23
Female (%)	62.9	61.9	65.7	67.5	59.3	64.6
Urban influence (%)	77.6	84.6	78.9	90.7	77.2	86.4
High School Degree only (%)	30.9	28.7	32.1	29.8	31.7	30.7
